# Immunogenicity and memory B-cell potency induced by an inactivated COVID-19 vaccine in pregnant women

**DOI:** 10.1186/s10020-025-01357-5

**Published:** 2025-09-26

**Authors:** Gui-Ping Wen, Yi-Zhen Wang, Min-Ming Wang, Wen-Rong Wang, Si-Ling Wang, Zheng Wang, Zi-Min Tang, Zhen-Yu Luo, Zi-Hao Chen, Jia-Yan Chen, Mei-Jiao Cai, Yun-Sheng Ge, Zi-Zheng Zheng, Yu-Lin Zhou

**Affiliations:** 1 Department of Central Laboratory, Fujian Key Clinical Specialty of Laboratory Medicine, Department of Obstetrics and Gynecology, Women and Children’s Hospital, School of Medicine, Xiamen University, Xiamen, Fujian 361102 China; 2 United Diagnostic and Research Center for Clinical Genetics, Women and Children’s Hospital, School of Medicine, School of Public Health, Xiamen University, Xiamen, Fujian 361102 China; 3 State Key Laboratory of Molecular Vaccinology and Molecular Diagnostics, National Institute of Diagnostics and Vaccine Development in Infectious Diseases, School of Public Health, Xiamen University, Xiamen, Fujian 361102 China; 4 Xiang An Biomedicine Laboratory, Xiamen, Fujian 361102 China; 5 Department of Aristogenesis, Department of Obstetrics and Gynecology, Women and Children’s Hospital, School of Medicine, Xiamen University, Xiamen, Fujian 361102 China; 6 NMPA Key Laboratory for Research and Evaluation of Infectious Disease Diagnostic Technology, School of Public Health, Xiamen University, Xiamen, Fujian 361102 China; 7 School of Pharmacy, Xiamen University, Xiamen, Fujian 361102 China

**Keywords:** Humoral immune response, Inactivated COVID-19 vaccine, Pregnancy, Memory B cell, SARS-CoV-2.

## Abstract

During pregnancy, profound immunological, hormonal, and metabolic adaptations occur to support fetal development. The impact of pregnancy on vaccine-induced immunity remains incompletely characterized, as previous studies have primarily focused on serological antibody levels but not immune memory. Immune memory is critical for vaccine effectiveness, but effect of pregnancy on immune memory remain unknown. In addition, the memory B cell response profile induced by inactivated coronavirus disease 2019 (COVID-19) vaccines in pregnant women remains unclear. This study comprehensively investigated the serological responses and memory B cell response induced by an inactivated COVID-19 vaccine in pregnant women. The results demonstrated that while pregnant women and non-pregnant women of childbearing age showed comparable serological antibody levels, vaccine-induced monoclonal antibodies (mAbs) from pregnant women exhibited significantly lower binding potency to severe acute respiratory syndrome coronavirus 2 (SARS-CoV-2) spike protein and its segments and weaker neutralizing potency and breadth than those from non-pregnant women. Vaccine-induced mAbs from pregnant women were derived predominantly from IGHV3-30, whereas those from non-pregnant women were derived diverse germline genes. Most of mAbs from pregnant women targeted the receptor-binding domain (RBD) (40.9%) and S2 domain (31.8%), whereas most of mAbs from non-pregnant women targeted the RBD (51.3%) and N-terminal domain (30.8%). These findings suggested that pregnancy may impair the potency of vaccine-induced memory B cells. These insights may be valuable for the development of vaccination strategies for pregnant women.

**Supplementary Information:**

The online version contains supplementary material available at 10.1186/s10020-025-01357-5.

## Introduction

Significant immune changes occur during pregnancy, enabling the mother to develop immune tolerance to the fetus and facilitating fetal growth. In addition to physiological and hormonal changes, susceptibility to particular infections and their severity are elevated [1, 2]. For example, during the 1918 influenza pandemic, the mortality rate among pregnant women reached as high as 27% [2]. During the 2009 influenza pandemic, pregnant women were approximately seven times more likely to suffer from severe influenza-associated disease and two times more likely to die than non-pregnant women were, and 5% of deaths due to this disease occurred in pregnant women [3]. With respect to malaria parasites, pregnant women are three times more likely to suffer from severe malaria than non-pregnant women are, with the median maternal mortality due to malaria in the Asia–Pacific region being 39% [2]. A similar pattern is observed for hepatitis E virus (HEV) infection, for which the mortality rate is approximately 1% in the general population but as high as 25% among pregnant women; furthermore, HEV infection in pregnant women is often accompanied by high rates of spontaneous abortion and stillbirth [4]. In the context of severe acute respiratory syndrome coronavirus 2 (SARS-CoV-2), pregnant women experience more severe disease and higher morbidity than non-pregnant women of reproductive age do [5, 6].

Vaccines are recognized as one of the greatest medical achievements, having successfully eradicated many infectious diseases [7, 8]. The eradication of smallpox is an exemplary examples of how vaccination eliminate a fatal disease and prevent millions of deaths [9][7]. Due to the global adoption of vaccination, the incidence rates of many childhood diseases, including polio and measles, have been dramatically reduced [7, 8, 10]. However, clinical research and development on vaccines for pregnant women are fraught with significant challenges. Various phases of clinical trials for vaccines are being conducted to determine vaccine safety, immunogenicity, and efficacy. Despite their critical need for these vaccines, pregnant women are often excluded from participating in these clinical trials because of safety concerns [11, 12]. Like almost all clinical trials, the trials of hepatitis E vaccine [13], human Papillomavirus vaccines [14, 15], and coronavirus disease 2019 (COVID-19) vaccines [12, 16] have excluded pregnant women from participating. Currently, only a limited number of vaccines, such as tetanus toxoid, reduced diphtheria toxoid, and acellular pertussis vaccine (Tdap) and inactivated influenza, are recommended for pregnant women [17, 18]. Despite the exclusion of pregnant women from COVID-19 vaccine clinical trials, existing observational data suggest that the benefits of vaccination outweigh the potential risks, leading some regulatory bodies to recommend COVID-19 vaccination for pregnant women [6, 19]. Nevertheless, policies regarding COVID-19 vaccination in pregnant women vary across countries [20]. Pregnant women were not recommended to receive COVID-19 vaccines in China and United Arab Emirates [20]. In contrast, pregnant women were recommended to receive COVID-19 vaccines in USA [21, 22], Brazil [23], Germany [24], and France [25].

There are many unanswered questions regarding the immunological changes that occur during pregnancy [2]. Whether pregnancy affects vaccine-induced immunity remains understudied. Researchers have conducted a series of studies to investigate the effects of pregnancy on vaccine-induced immunity [17, 21, 26–34]. Most of these studies were focused on determining the immunogenicity of vaccines, through the analysis of serum antibody levels [21, 26, 27, 29–31]. T-cell response, the glycosylation and Fc functionality of antibodies have been examined in several studies [28, 30, 32, 33]. Huygen et al. [28] demonstrated that vaccine-specific Th1 type cellular immune responses were impaired during pregnancy. Antibody effector functions were moderately reduced or delayed during pregnancy [32, 33]. Seasonal influenza vaccine-induced antibodies in pregnant women exhibited differential Fc/Fab glycosylation characterized by increased sialylation and galactosylation compared with those in non-pregnant women [33]. Additionally, Kay et al. [34] showed that pregnant women displayed differential circulating plasmablast production compared to non-pregnant women following influenza vaccination. Immune memory plays a critical role in vaccine efficacy, but research on the impact of pregnancy on immune memory, especially memory B cells, is still limited. Notably, significant differences have been noted in the RSV-specific memory B cells between adults and infants infected with RSV [35, 36].

COVID-19 vaccines have been crucial in controlling the spread of SARS-CoV-2 infection, especially in the early stages of the pandemic [37]. The SARS-CoV-2 genome primarily encodes four structural proteins, with the spike (S) protein playing a key role in virus entry into cells and being a primary target for antibody responses and vaccine development [38]. The S protein is composed of two subdomains: S1 and S2. The former mainly contains the receptor-binding domain (RBD) and the N-terminal domain (NTD), with the RBD being the primary target for neutralizing monoclonal antibodies (mAbs) [38, 39]. Inactivated COVID-19 vaccines are highly safe and effective and are deployed globally to prevent COVID-19 [40]. Previous studies have also shown that inactivated vaccines are safe [19, 41–43], and effective at inducing neutralizing antibodies in pregnant women [41, 43], with a positive rate of neutralizing antibodies ranging from 39.5 to 62.3% after two doses. These inactivated vaccines seem to effectively prevent symptomatic COVID-19 and accelerate the clearance of SARS-CoV-2 [23, 42]. However, the detailed characteristics of memory B-cell responses and the specific mAbs induced by these vaccines in pregnant women remain largely unexplored. Addressing this question is essential for providing insights into the mechanisms underlying the protection of vaccines and informing their effectiveness.

The aim of this study was to perform an in-depth analysis of the serological immune responses and memory B-cell potency induced by an inactivated COVID-19 vaccine in pregnant and non-pregnant women of childbearing age. We thoroughly explored the genetic, functional, and epitopic characteristics of SARS-CoV-2 S-specific mAbs generated postvaccination in both groups. Our findings will be informative for optimizing and updating vaccine design and immunization strategies for pregnant women.

## Materials and methods

### Study participants and sample collection

Fifteen women who received two doses of the inactivated COVID-19 vaccine CoronaVac (Sinovac, Beijing, China) and had no history of SARS-CoV-2 infection were recruited with informed consent. The two vaccine doses were administered at intervals of 21 to 38 days. All 15 participants were routinely tested for SARS-CoV-2 by real-time PCR and tested negative. Due to the effective intervention and control measures of the COVID-19 pandemic, less than 300 laboratory-confirmed cases occurred before December 2022 in the study region with more than 5.2 million people [44]. All participants have no underlying diseases, such as immune disorders and cancer, and had not received blood products within the preceding year. Among them, four pregnant women received the vaccine inadvertently in their first trimester, and the remaining 11 non-pregnant women of childbearing age received the same vaccine regimen (Table S1). Plasma samples and some peripheral blood mononuclear cells (PBMCs) were collected from participants within 90 days after their second dose. Samples were collected between August 2021 and November 2021. This study was designed in accordance with the principles of the Declaration of Helsinki and approved by the Ethics Committee of the Women and Children’s Hospital, School of Medicine, Xiamen University.

### Protein expression and purification

The S protein of SARS-CoV-2 was recombinantly expressed and purified as previously described [45]. The S protein includes a ‘GSAS’ substitution at the furin cleavage site and an N1192M substitution. The recombinant S protein was produced in stable cell lines and purified using Q-FF Sepharose ion-exchange chromatography (Cytiva). Other SARS-CoV-2 S1, RBD, NTD, and S2 proteins were acquired from Sino Biological (Beijing, China).

### Indirect enzyme-linked immunosorbent assay

The binding activities of the plasma and mAbs were determined using an indirect enzyme-linked immunosorbent assay (ELISA) as described previously [46]. The microplates were precoated with 100 ng of S, S1, RBD, NTD, and S2 protein per well. One hundred microliters of serially diluted plasma or mabs were added to each well of the microplates and incubated at 37 °C for 30 min. Plasma samples were initially diluted to 1:10, followed by five subsequent serial 10-fold dilutions. Following five washes with PBS containing 0.05% Tween-20 (PBST), 100 µL of horseradish peroxidase (HRP)-conjugated goat anti-human IgG, IgM, or total secondary antibody was added. After another 30-min incubation, the microplates were washed five times, and 100 µL of tetramethylbenzidine (TMB) was added. The reaction was stopped after 15 min at 37 °C with 50 µL of 2 M H_2_SO_4_ and the optical density (OD) was read at 450 nm with a reference wavelength of 630 nm. For plasma, the binding activities are presented as the half-maximal effective dilution (ED_50_). For mAbs, the binding activities are presented as the half-maximal effective concentration (EC_50_). Both the ED_50_ and EC_50_ values were calculated using the 4-parameter logistic (4PL) regression in GraphPad Prism.

### Pseudovirus neutralization assay

The neutralizing activity of plasma and mAbs against SARS-CoV-2 strains was assessed using a lentivirus-based pseudovirus neutralization assay as previously described [39, 47]. The SARS-CoV-2 pseudoviruses, including D614G, Alpha, Beta, Gamma, Delta, and Omicron sublineages BA.1, BA.2.12.1, BA.4/5, BF.7, BQ1.1, XBB, and EG.1, were produced as described previously [39]. Serially diluted plasma or mabs were preincubated with pseudoviruses at 37 °C for 1 h. The mixture was subsequently incubated with H1299-ACE2hR cells that had been seeded in 96-well cell culture plates with optically clear bottoms (PerkinElmer). The fluorescence images of the cells were captured 48 h postinfection with an Opera Phenix high-content imaging system (PerkinElmer) and analyzed with Columbus Software version 2.5.0 (PerkinElmer). For plasma, the neutralizing titers were quantified as the maximum dilution required to achieve infection inhibition by 50% (ID_50_). An ID_50_ ≥ 20 was set as the cutoff value for the presence of neutralizing antibodies in the plasma. For mAbs, the neutralizing potency was quantified as the half-maximal inhibitory concentration (IC_50_). Both the ID_50_ and IC_50_ values were calculated using the 4PL regression in GraphPad Prism.

### Flow cytometry and single B-cell sorting

SARS-CoV-2 S-specific B cells were isolated as previously reported [46, 48]. Briefly, PBMCs from five individuals were stained with a cocktail of live/dead-Aqua dead cell stain kit (Invitrogen), CD3-PE-Cy7 (BD Biosciences), CD19-BV786 (BD Biosciences), CD27-BV650 (BD Biosciences), anti-human IgM-PerCP-Cy5.5 (BD Biosciences), anti-human IgG-BV421 (BD Biosciences), S-FITC and biotinylated S on ice for 30 min, followed by streptavidin-APC (SA-APC) (Molecular Probes) for an additional 30 min. Single IgG + memory B cells (identified as live + CD3-CD19 + CD27 + IgM-IgG+) positive for the S protein were individually sorted into the wells of 96-well plates containing lysis buffer on an Aria III sorter (BD Biosciences).

### Antibody gene amplification, cloning, and expression

Human antibody heavy and light chain variable genes (IgH, Igλ, and Igκ) were amplified by nested reverse transcription PCR as described previously [46, 48, 49]. The sequences were analyzed via the IMGT V-quest webserver (https://www.imgt.org/IMGT_vquest). Paired heavy and light chains were cloned and inserted into expression vectors carrying constant regions of the human IgG1 heavy chain and light chain. The constructed plasmids were transiently transfected into ExpiCHO-S cells (Thermo Scientific) according to the manufacturer’s protocol. The supernatants were collected 14 days post transfection, and the antibodies were purified with a protein-A affinity chromatography column (Cytiva).

### Competitive ELISA

The epitopes of the mAbs that recognized the RBD were determined as previously reported [46]. In brief, unlabeled mAbs or PBS (50 µL) were added to RBD-coated microplates and incubated at 37 °C for 30 min. Subsequently, 50 µL of HRP-conjugated Mabs was added directly to the microplates at selected dilutions. The dilution factor for each HRP-conjugated Mab was optimized individually due to variations in binding affinity to the RBD. The selected dilution for each HRP-conjugated mAb refers to the concentration that yields OD values of approximately 1.5 in the presence of PBS. After a 30-min incubation, the microplates were rinsed five times, and the color was developed with TMB substrate. The blocking efficiency (%) was calculated by comparing the OD values in the presence and absence of competitor mAbs.

### Statistical analysis

Statistical analyses were conducted with GraphPad Prism and R software (version 4.2.2). Differences were assessed using the Mann‒Whitney U test or unpaired t test. Correlations were explored using Spearman rank correlation analysis. mAbs that failed to neutralize SARS-CoV-2 were excluded from the correlation analysis. because their IC_50_ values could not be accurately determined. p values were calculated from a two-tailed test with significance thresholds set at < 0.05. ns: no significant difference; *: *p* < 0.05; **: *p* < 0.01; ***: *p* < 0.001; ****: *p* < 0.0001.

## Results

### SARS-CoV-2-specific antibody levels in pregnant women and non-pregnant women

**Fig. 1:**
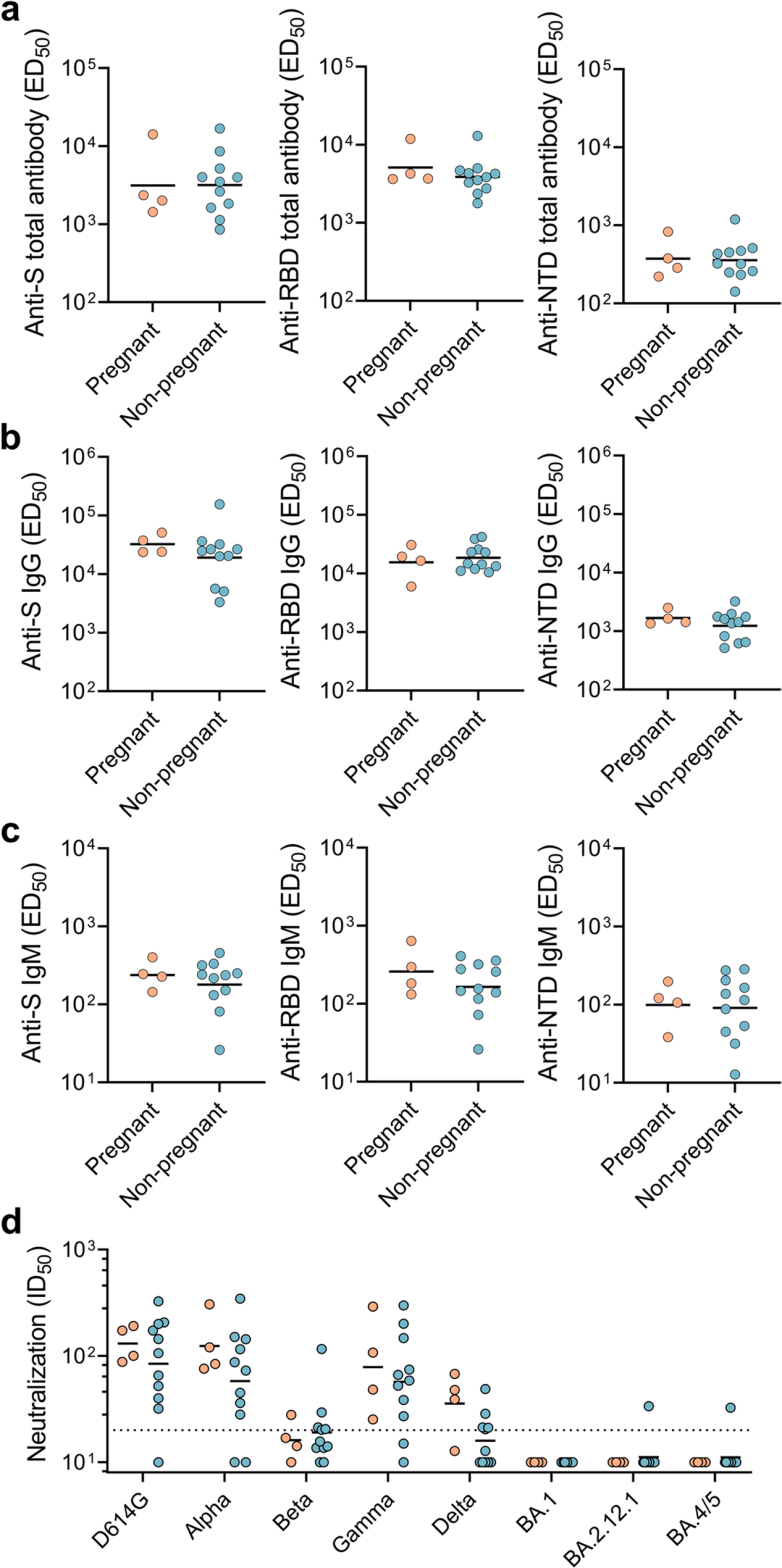
Severe acute respiratory syndrome coronavirus 2 (SARS-CoV-2)-specific humoral response in participants**. a** Levels of total antibody (half-maximal effective dilution, ED_50_) against the spike (S), receptor-binding domain (RBD), and N-terminal domain (NTD) proteins of SARS-CoV-2 in plasma. **b** Levels of IgG antibody (ED_50_) against the S, RBD, and NTD proteins of SARS-CoV-2 in plasma. **c** Levels of IgM antibody (ED_50_) against the S, RBD, and NTD proteins of SARS-CoV-2 in plasma. **d** Plasma neutralizing activity (maximum dilution required to achieve infection inhibition by 50%, ID_50_) against SARS-CoV-2 D614G, Alpha, Beta, Gamma, Delta, and Omicron sublineages BA.1, BA.2.12.1, and BA.4/5. The cutoff value for the neutralizing activity assay was set at ID_50_ ≥ 20. The difference was calculated via an unpaired t test after logarithmic transformation. No significant differences were observed in **(a)** to **(d)**

A total of 15 women were enrolled, including 4 pregnant women and 11 non-pregnant women (Table**S1**). Plasma antibody reactivity against the SARS-CoV-2 S protein and its segments (S1, S2, RBD, and NTD) was measured using ELISA. Overall, the levels of total antibody, IgM, and IgG in pregnant women were similar to those in non-pregnant women (Fig. 1a, b and c, and Fig. S1). The IgG responses to these viral proteins were much greater than those of IgM in both groups, suggesting that the dominant plasma antibody response was that of IgG. Notably, the levels of total antibody and IgG against the RBD were higher than those against the NTD and S2 in both pregnant women and non-pregnant women (Fig**. **S2), suggesting that the RBD is the main immunogenic component of the virus.

We next evaluated the plasma neutralizing activity against SARS-CoV-2 variants (D614G, Alpha, Beta, Gamma, Delta, and Omicron sublineages BA.1, BA.2.12.1, and BA.4/5) using a pseudotyped lentivirus-based neutralization assay. Neutralizing activity against the D614G, Alpha, and Gamma variants was detected in the majority of vaccine recipients (≥ 86.7%) (Fig. 1d and Table S1). The levels of neutralizing antibodies against D614G, Beta, Gamma, and Omicron sublineages in pregnant women were comparable to those in non-pregnant women (Fig. 1d). While neutralizing antibody levels against Alpha seem to be higher in pregnant women than those in non-pregnant women, the difference was not significant (*p* = 0.2299, t = 1.260). All individuals exhibited limited to no neutralizing activity against any Omicron sublineage (Fig. 1d andTable**S1**), which aligns with observations from previous studies [40, 50, 51]. Plasma neutralizing activity against D614G was significantly correlated with the anti-RBD total antibody (*r* = 0.5464, *p* = 0.0377) and anti-RBD IgG titers (*r* = 0.6500, *p* = 0.0105) but not with anti-NTD antibody levels or anti-S2 antibody levels (*p* > 0.05 for each) (Fig**. **S3). Similar trends were observed both in pregnant women and non-pregnant women (Fig**. **S4 and Fig**. **S5**)**. These findings confirmed that the RBD plays a key role in the neutralizing antibody response, which is consistent with previous studies [38, 39]. Taken together, the inactivated COVID-19 vaccine generated a robust humoral immune response in pregnant women, with immunogenicity similar to that observed in non-pregnant women.

### SARS-CoV-2 S-specific memory B-cell repertoire in pregnant women and non-pregnant women

**Fig. 2:**
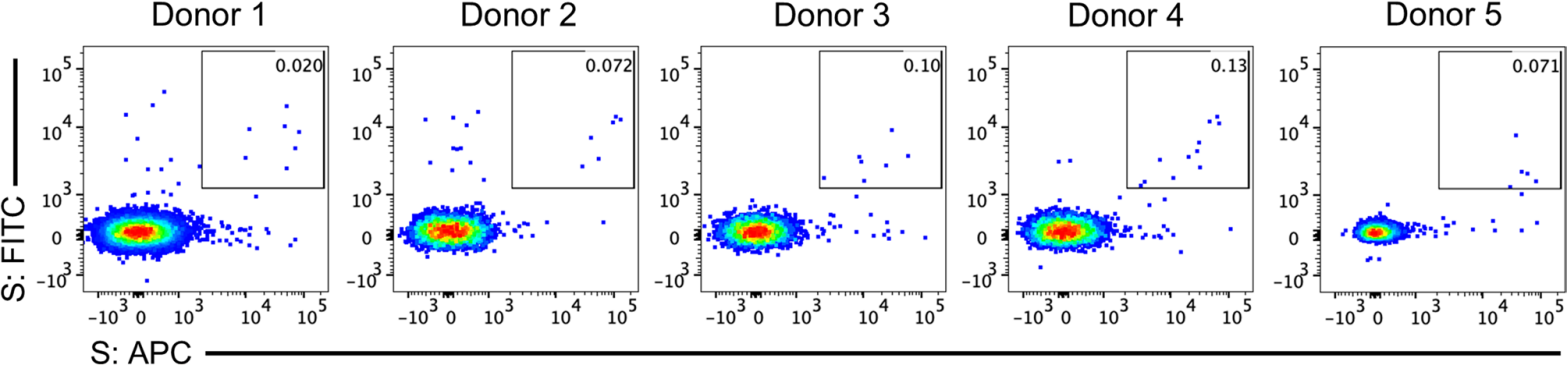
Percentages of severe acute respiratory syndrome coronavirus 2 (SARS-CoV-2) spike (S)-specific IgG + memory B cells in five individuals. Donors 1 and 2 were pregnant women, while donors 3, 4 and 5 were non-pregnant women of childbearing age. The values in the upper right corner of each panel represent the proportion of S-specific IgG + memory B cells for each participant. Total IgG + memory B cell counts were about 34,000 in donor 1, 8300 in donor 2 and donor 4, and 7000 in donor 3 and donor 5. In these five individuals, S-specific cells comprised 0.020–0.13% of total IgG + memory B cells

To further explore inactivated COVID-19 vaccine-induced antibody responses at the molecular level and identify memory B cells that produce anti-S antibodies, two pregnant and three non-pregnant women with sufficient PBMCs were selected for detailed analysis (Table S2). Flow cytometry was used to isolate individual B cells that bound to the S protein (Fig**. **S6). The results indicated that the frequency of S-specific memory B cells ranged from 0.02 to 0.072% in pregnant women and from 0.071 to 0.13% in non-pregnant women (Fig. 2). To determine whether there were differences between the groups in terms of the potency of vaccine-induced specific memory B cells, these S-specific memory B cells were individually isolated. In total, 22 SARS-CoV-2 S-specific Mabs were obtained from pregnant women and 39 were obtained from non-pregnant women (Table S2).

### Genetic characteristics of SARS-CoV-2 S-specific mAbs in pregnant women and non-pregnant women

**Fig. 3:**
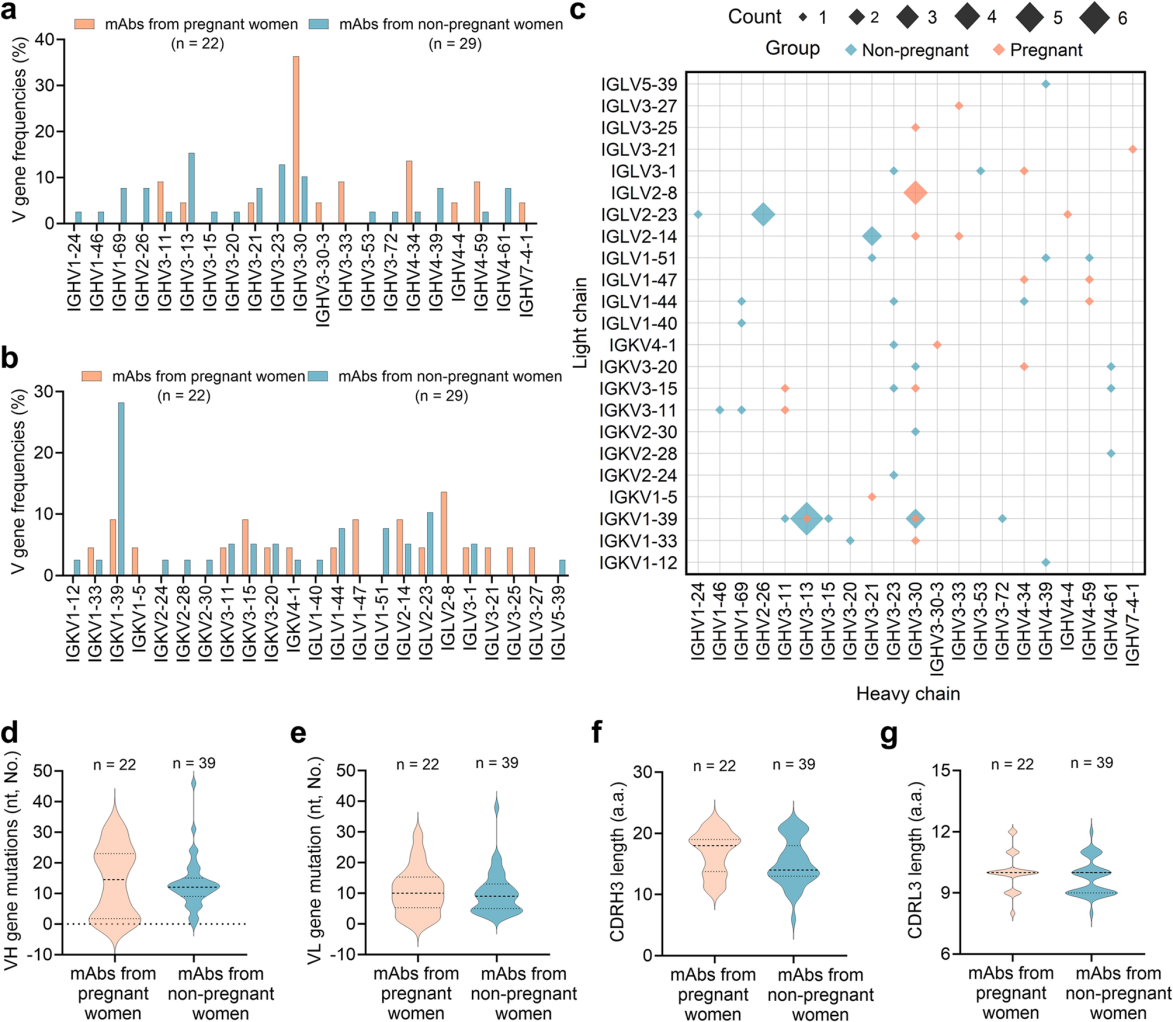
Characteristics of the variable regions of severe acute respiratory syndrome coronavirus 2 (SARS-CoV-2) spike-specific monoclonal antibodies isolated from pregnant women and non-pregnant women.** a** Distribution of variable (V) genes among heavy-chain (VH) germline genes. **b** Distribution of the V gene of light-chain (VL) germline genes. **c** Profiles of VH (X-axis) and VL (Y-axis) germline genes. The size of each point corresponds to the number of heavy and light chain pairs in the repertoire. The different colors represent different participant groups. **d** Distribution of nucleotide mutations in the VH gene. **e** Distribution of nucleotide mutations in the VL gene. **f** Distribution of complementarity-determining region 3 (CDR3) lengths of the heavy chain (CDRH3). **g** Distribution of the CDR3 lengths of the light chain (CDRL3). “a.a.” refers to amino acids. Differences in (**d**) to (**g**) were determined using an unpaired t test. No significant differences were observed. A total of 22 mabs were isolated from pregnant women and 39 mAbs from non-pregnant women

The genetic characteristics of 22 S-specific Mabs from pregnant women and 39 mAbs from non-pregnant women were analyzed. In terms of V gene usage, a diverse range of germlines genes was utilized to generate S-specific mAbs in both pregnant and non-pregnant women (Fig. 3). Some differences in the representation of IGHV, IGKV and IGLV genes were observed between the two groups. IGHV3-30 and IGLV2-8 were most abundant in pregnant women, accounting for 36.4% and 13.6% of heavy chains and light chains, respectively (Fig. 3a and Fig. 3b). The IGHV3-30-derived heavy chains paired with various light chains to form antibodies in pregnant women (Fig. 3c). In contrast, IGHV3-13, IGHV3-23, and IGHV3-30 were most prevalent in non-pregnant women, accounting for 15.4%, 12.8%, and 10.3% of heavy chains, respectively. IGKV1-39 was most abundant in non-pregnant women, Making up 28.2% of the light chains (Fig. 3b). All IGHV3-13-derived heavy chains paired with IGKV1-39-derived light chains to form antibodies in pregnant and non-pregnant women (Fig. 3c).

We further explored the V gene somatic hypermutation (SHM) levels and the complementarity determining region (CDR3) length for the mAbs. Consistent with previous reports on mAbs generated from SARS-CoV-2 infection and the COVID-19 vaccine [40, 52, 53], the mAbs exhibited a relatively low degree of SHM. The extent of SHM for the V genes of heavy and light chains was comparable between pregnant and non-pregnant women (Fig. 3d and Fig. 3e). The average number of nucleotide substitutions in V gene of heavy chain (VH) was 14.0 in pregnant women and 13.2 in non-pregnant women (Fig. 3d), similar to anti-respiratory syncytial virus (RSV) mAbs, anti-influenza mAbs and anti-HEV mAbs [35, 49, 54]. The CDR3 lengths of the heavy and light chains of mAbs were also comparable between pregnant and non-pregnant women, with no significant difference (Fig. 3f and Fig. 3g). Nearly half (43.6%) of the mAbs in non-pregnant women were IGHJ4-derived antibodies (Fig**. S7**). These results indicated subtle differences in the genetic characteristics of SARS-CoV-2 S-specific mAbs isolated from pregnant and non-pregnant women.

### Functional activities of vaccine-induced mAbs from pregnant and non-pregnant women

**Fig. 4:**
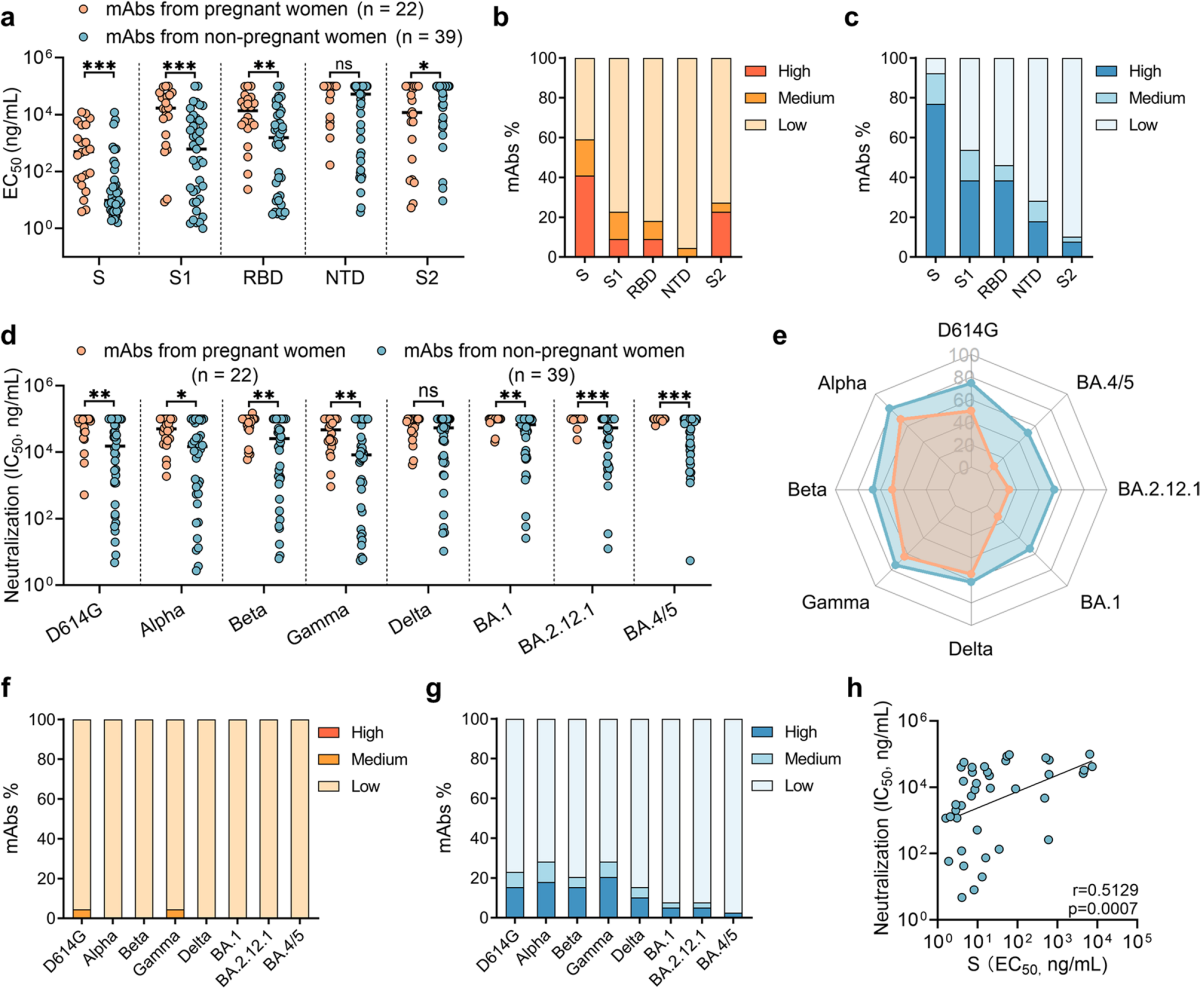
Functional activities of severe acute respiratory syndrome coronavirus 2 (SARS-CoV-2) spike (S)-specific monoclonal antibodies (mAbs) isolated from pregnant women and non-pregnant women**. a** Antibody-binding activity (half-maximal effective concentration, EC_50_) of the SARS-CoV-2 S, S1, receptor-binding domain (RBD), N-terminal domain (NTD), and S2 protein. Each dots represent a single mAb. **b** Percentage of mAbs with high binding activity (EC_50_ < 100 ng/mL), medium binding activity (EC_50_ between 100 ng/mL and 1 µg/mL), and low binding activity (EC_50_ > 1 µg/mL) in pregnant women. **c** Percentage of mAbs with high binding activity (EC_50_ < 100 ng/mL), medium binding activity (EC_50_ between 100 ng/mL and 1 µg/mL), and low binding activity (EC_50_ > 1 µg/mL) in non-pregnant women. **d** Antibody-neutralizing activity (half-maximal inhibitory concentration, IC_50_) against SARS-CoV-2 D614G, Alpha, Beta, Gamma, Delta, BA.1, BA.2.12.1, and BA.4/5. Each dots represent a single mAb. **e** Radar map of the neutralizing activity positive rate against SARS-CoV-2 D614G, Alpha, Beta, Gamma, Delta, BA.1, BA.2.12.1, and BA.4/5. Blue indicates non-pregnant women; orange indicates pregnant women. **f** Percentages of mAbs with high neutralizing activity (IC_50_ < 100 ng/mL), medium neutralizing activity (IC_50_ between 100 ng/mL and 1 µg/mL), low neutralizing activity (IC_50_ > 1 µg/mL) in pregnant women. **g** Percentages of mAbs with high neutralizing activity (IC_50_ < 100 ng/mL), medium neutralizing activity (IC_50_ between 100 ng/mL and 1 µg/mL), and low neutralizing activity (IC_50_ > 1 µg/mL) in non-pregnant women. **(h)** Correlation between binding activity and neutralizing activity. p values and r values were derived from the Spearman rank test in (**h**). Differences in **(a)** and **(d)** were determined using the Mann‒Whitney U test. ns, no significant difference; *p < 0.05; **p < 0.01; ***p < 0.001; ****p < 0.0001. A total of 22 Mabs were isolated from pregnant women and 39 mAbs from non-pregnant women

The functional activities of 22 S-specific Mabs isolated from pregnant women and 39 mAbs isolated from non-pregnant women were subsequently analyzed. We used ELISAs to assess the binding activity of these mAbs to the S, S1, S2, RBD, and NTD proteins. We found that the mAbs from non-pregnant women exhibited significantly greater binding activities to the SARS-CoV-2 S protein than those from pregnant women did (*p* = 0.0001), with median EC_50_ values of 9.7 ng/mL versus 496.8 ng/mL, respectively (Fig. 4a). When analyzed on a per-participant basis, the mean binding activity of mAbs from each pregnant woman to the S and S1 proteins were also lower than those in each non-pregnant woman (Fig**. **S8a). Compared with pregnant women, non-pregnant women had a greater proportion of mAbs with high binding activity (EC_50_ < 100 ng/mL) (76.9% vs. 40.9%) and a lower proportion of mAbs with weak binding activity (EC_50_ > 1000 ng/mL) (7.7% vs. 40.9%) (Fig. 4b and Fig. 4c). Additionally, mAbs from non-pregnant women presented significantly greater binding activities to the RBD, S1, and S2 than those from pregnant women did (*p* < 0.05 for each) (Fig. 4a).

We further used a pseudotyped SARS-CoV-2 neutralization assay to evaluate the neutralizing activity of all mAbs. Consistent with the binding results, mAbs from pregnant women exhibited significantly lower neutralizing activities against all tested SARS-CoV-2 strains except Delta than those from non-pregnant women did (*p* < 0.05 for each) (Fig. 4d). When analyzed on a per-participant basis, the mean neutralizing activity of mAbs from each pregnant woman against D614G, BA.2.12.1, and BA.4/5 was lower than those from each non-pregnant woman (Fig**. **S8b). Overall, the percentage of mAbs that were able to neutralize SARS-CoV-2 variants was greater in non-pregnant women than in pregnant women (Fig. 4e). Most mAbs isolated from pregnant women were incapable of neutralizing Omicron sublineages (Fig. 4e). Moreover, a greater percentage of mAbs isolated from non-pregnant women than from pregnant women presented potent neutralizing activity (IC_50_ < 100 ng/mL) (Fig. 4f and Fig. 4g). Ten (25.6%) Mabs from non-pregnant women were able to neutralize at least 7 of the 8 strains tested, with an IC_50_ of < 10,000 ng/mL, but none of the Mabs was observed in pregnant women. These 10 mAbs were further tested for their neutralizing activity against other Omicron sublineages (including BF.7, BQ1.1, XBB, and EG.1), and nine of them showed neutralizing activity against these four variants (Fig**. **S9). Further analysis revealed a significant correlation between S binding activity and neutralization activity (*r* = 0.5129, *p* = 0.0007) (Fig. 4h) but not between SHM and neutralization activity (Fig**. **S10), indicating that extensive maturation is not required for effective virus neutralization, which is in line with observations in SARS-CoV-2-infected patients [52]. In summary, neutralizing antibodies against various SARS-CoV-2 variants could be induced in individuals immunized with the inactivated COVID-19 vaccine, albeit with compromised potency. Compared with those in pregnant women, the antibodies expressed by vaccine- specific memory B cells in non-pregnant women exhibited significantly greater functional activity.

### Vaccine-induced mAbs from pregnant and non-pregnant women recognize different domains or epitopes

**Fig. 5:**
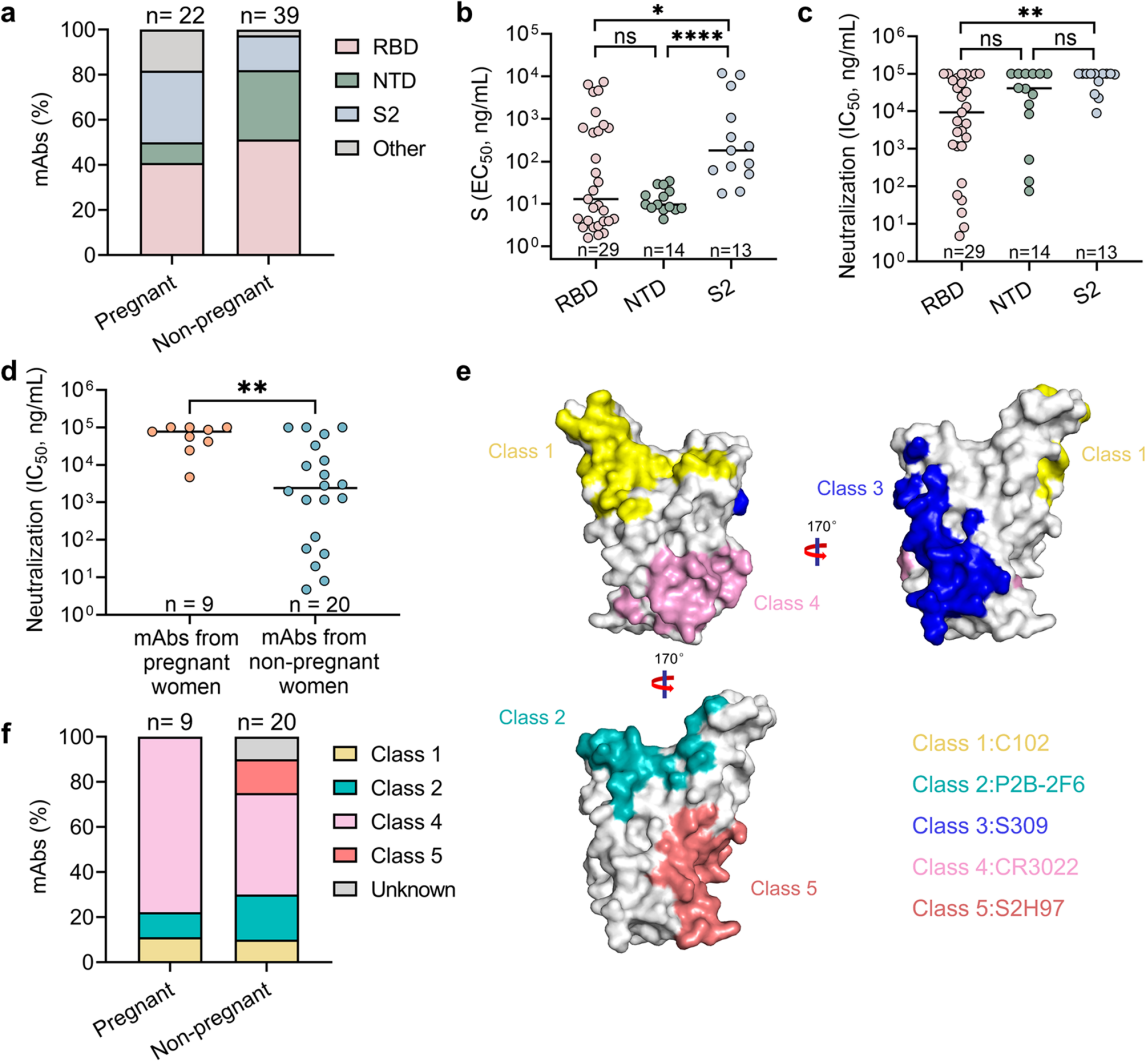
Epitope-function mapping of severe acute respiratory syndrome coronavirus 2 (SARS-CoV-2) spike (S)-specific monoclonal antibodies (mAbs)**. a** Percentages of mAbs targeting the receptor-binding domain (RBD), N-terminal domain (NTD), and S2 in pregnant and non-pregnant women. Twenty-two mabs were isolated from pregnant women and 39 were from non-pregnant women. **b** Antibody binding activity (half-maximal effective concentration, EC_50_) plotted against the RBD, NTD, and S2. Each dot indicates a single mAb. **c** Antibody neutralizing activity (half-maximal inhibitory concentration, IC_50_) plotted against the RBD, NTD, and S2. Each dot indicates a single mAb. **d** Neutralizing activity (IC_50_) of RBD-directed mAbs in pregnant and non-pregnant women. Each dot indicates a single mAb. **e** Spatial locations of the five subtypes (classes 1 to 5) on the SARS-CoV-2 RBD surface. Representative Mabs for classes 1 to 5 include C102, P2B-2F6, S309, COVA1-16 and S2H97. The color-coding scheme is as follows: C102 (yellow), P2B-2F6 (teal), S309 (blue), COVA1-16 (pink), and S2H97 (salmon). **f** Composition of Mabs directed to classes 1 to 5 on the SARS-CoV-2 RBD. Differences in **(b)** to **(d)** were determined using the Mann-Whitney U test. ns, no significant difference; *p < 0.05; **p < 0.01; ***p < 0.001. ****p < 0.0001

Given the differences in the functional activities of vaccine-specific mAbs from pregnant and non-pregnant women, we further compared whether mAbs isolated from both groups recognized the same domains or epitopes. First, the mAbs were categorized on the basis of their recognition of the RBD, NTD, S2, and other domains. The majority of mAbs from pregnant women (40.9%) and non-pregnant women (51.3%) targeted the RBD (Fig. 5a), indicating that the RBD is the main target of the antibody response induced by the inactivated vaccine. Additionally, a significant percentage of mAbs from pregnant women (31.8%) recognized S2, whereas a considerable percentage of mAbs from non-pregnant women (30.8%) recognized the NTD. mAbs targeting the RBD showed significantly greater binding and neutralizing activities than those targeting the S2 domain did (Fig. 5b and Fig. 5c). However, when analyzed on a per-participant basis, the mean neutralizing activity of RBD-, NTD-, and S2-targeting mAbs were comparable across participants (Fig**. **S11). IGHV3-13 and IGHV3-30 accounted for 24.1% and 20.7%, respectively, of the mAbs that recognized the RBD (Fig**. **S12a). None of the mAbs recognizing the NTD were derived from IGHV3-13 or IGHV3-30; most of them were derived from IGHV3-23 and IGHV2-26 (Fig**. **S12b).

Despite targeting the RBD, mAbs from pregnant women and non-pregnant women exhibited significant differences in their neutralization capabilities and binding activities (Fig. 5d and Fig**. **S13). When analyzed on a per-participant basis, the mean neutralizing activity of RBD-targeting mAbs from each pregnant woman was lower than that from each non-pregnant woman (Fig**. **S14). We further analyzed the epitopes recognized by these mAbs through a cross-blocking assay with antibodies classified into five structurally defined epitope classes (C102 for class 1, P2B-2F6 for class 2, S309 for class 3, CR3022 for class 4, and S2H97 for class 5) (Fig. 5e), as previously reported [55]. Overall, the majority of the mAbs targeted four classes (class 1, class 2, class 4, and class 5) (Fig. 5f). Most Mabs isolated from both pregnant and non-pregnant women belonged to class 4 (77.8% vs. 45.0%) (Fig. 5f). Approximately 10% of the Mabs isolated from pregnant women and non-pregnant women belonged to class 1, and 11.1% and 20.0% belonged to class 2. In addition, 15.0% of the Mabs from non-pregnant women belonged to class 5. Collectively, these findings demonstrate that the inactivated vaccine could induce antibodies that target distinct domains or epitopes, similar to those observed following natural infection. There were certain differences in the recognition of S-specific antibodies induced by the inactivated vaccine between pregnant and non-pregnant women.

## Discussion

The impact of pregnancy on the quality of vaccine-induced immune responses remains an unresolved question. Researchers have conducted numerous studies on vaccine-induced immunity in pregnant women, but the focus of these studies was primarily on immunogenicity [21, 26–34]. Pregnant women mount adequate immunological responses to vaccines against several pathogens [2, 17]. Pregnant women who receive the influenza vaccine develop protective antibody levels comparable to those of non-pregnant women [26, 27]. However, data on the effects of pregnancy on antibody titers following Tdap vaccination are variable [28, 29]. The focus of recent research on the immunogenicity of COVID-19 vaccines in pregnant women has been mainly on mRNA vaccines, with results indicating that antibody levels following mRNA COVID-19 vaccination are comparable between pregnant and non-pregnant women [21, 31]. Our study revealed that pregnant women generated robust antibody responses to the inactivated COVID-19 vaccine, achieving comparable vaccine-specific antibody levels and neutralizing antibody titers to those of non-pregnant women. The neutralizing antibody positivity rate was 93.3%, which was higher than that reported in previous studies [41, 43]. These findings help to elucidate the effectiveness of inactivated vaccines in pregnant women [23, 42].

Immune memory is crucial for vaccine effectiveness [56]. In this study, we systematically analyzed the nature of vaccine-induced specific memory B cells. Approximately 50.0% of the mabs expressed by memory B cells in pregnant women and 74.4% in non-pregnant women exhibited pseudovirus neutralizing activity. These rates were higher than the 17% neutralization rate observed in individuals receiving an inactivated COVID-19 vaccine [40] and were similar to those reported in COVID-19 patients (23%−81%) [52] [46]. Our findings indicated that the binding and neutralizing activities of mAbs produced by pregnant women were significantly lower than those produced by non-pregnant women. This finding aligns with previous observations that symptomatic COVID-19 protection efficacy was 41% in pregnant women after two doses of the inactivated vaccine in Brazil, whereas it was 53% in the general population [23].

The most commonly used heavy chain V gene families in humans are IGHV3 and IGHV4 [57]. Previous studies have shown that SARS-CoV-2 infection-induced S-specific or RBD-specific mAbs, including IGHV3-30, IGHV3-53, and IGHV3-30-3, primarily originate from IGHV3 [52, 53, 56, 58]. In individuals receiving the inactivated COVID-19 vaccine, IGHV3-30 and IGHV3-53 were highly overrepresented in the RBD-binding mAbs [40]. In our study, IGHV3-30 and IGHV3-13 were the most common S-specific mAbs from pregnant and non-pregnant individuals, respectively, who received the inactivated COVID-19 vaccine. These observations suggest potential genetic differences in vaccine-induced mAbs between these groups. This finding was similar to previous findings in RSV infection [35, 36], where significant differences in the genetic characteristics of RSV-specific antibody repertoires between adults and infants have been noted, with primarily IGHV1-69 and IGHV1-18 being used in adults and predominantly IGHV3-21 being used in infants.

Consistent with a previous study [40], this study showed that the inactivated COVID-19 vaccine could induce antibodies targeting various epitopes similar to those induced by SARS-CoV-2 infection. Specifically, 40.9% of S-specific mabs induced by the inactivated vaccine in pregnant women and 51.3% in non-pregnant women recognized the RBD, which was comparable to the findings in SARS-CoV-2-infected individuals (38.1%) [52]. The predominant RBD-binding mabs in both pregnant and non-pregnant women belong to class 4, differing from those in individuals receiving mRNA vaccines, where RBD-binding antibodies primarily belong to classes 1 and 2 [58]. In pregnant women, mAbs recognizing the S2 subunit were more prevalent, whereas in non-pregnant women, mAbs recognizing the NTD were more common. Additionally, approximately 15% of RBD-recognizing mAbs in non-pregnant women belonged to class 5, and these mAbs were not found in pregnant women. These results suggest potential differences in the recognition domains or epitopes of vaccine-induced mAbs between pregnant and non-pregnant women, similar to observations in RSV infections [35, 36], where mAbs in adults primarily recognized Sites Ø, II/III, and V, whereas mAbs in infants recognized Site III.

The findings of this study suggested that although vaccine-induced specific antibody levels were similar between pregnant and non-pregnant women, there were potential differences in the potency of memory B cells. Similar phenomena have been observed in other studies, where pregnant and non-pregnant women demonstrated differences in certain aspects despite comparable serological antibody levels [28, 32–34]. Kay et al. [34] reported that pregnant and non-pregnant women had similar robust serological immune responses after receiving an inactivated influenza vaccine, but differed in the induction and production of circulating plasmablasts. Huygen et al. [28] reported that although vaccine-specific antibodies increased to the same extent in pregnant and non-pregnant women after Tdap vaccination, the stimulation of vaccine-specific Th1-type cellular immune responses was impaired during pregnancy. Atyeo et al. [32] reported that vaccine-specific titers in pregnant and non-pregnant women were comparable after mRNA COVID-19 vaccination, but Fc receptor binding and antibody effector functions were induced with delayed kinetics in pregnant women compared with non-pregnant women. Significant differences in FcγR1 binding and Fc-functionality between pregnant women and non-pregnant women were observed following influenza vaccination, although serological responses remained largely intact during pregnancy [33]. During pregnancy, immune alterations, along with other profound hormonal, metabolic, and physiological changes may affect vaccine-induced immunity.

B cells are the primary antibody-producing and antigen-presenting cells and possess immunomodulatory functions [59]. Several studies have showed that B cells may behave differently during pregnancy [1, 18, 59–63]. T follicular helper (TFH) cells can induce B cell activation, a pivotal event for generating T cell-dependent B cell responses [18, 64]. The expansion of TFH cells during pregnancy has a positive effect on B cell responses and humoral immunity [18, 65]. However, estrogen inhibits B cell lymphocytes generation, leading to reduced peripheral blood B cell in pregnant women during pregnancy [60, 61]. Additionally, some studies have showed that B cell functions and activities were attenuated during pregnancy [1, 62, 63]. Chen et al. [62] showed that genes associated with B cell functions, including “B cell receptor signaling pathway” and “B cell activation”, were downregulated in the peripheral blood of pregnant women compared with non-pregnant women. Recently, Liu et al. [63] demonstrated that CD40 signaling related to B-cell responses was reduced during pregnancy. Previous studies have provided conflicting results regarding immunoglobulin (Ig) levels during pregnancy [1]. Notably, changes in IgG Fc domain glycosylation occur during pregnancy and IgG Fc domain glycosylation have immune regulatory functions and modulate IgG effector functions [1, 66]. Currently, our understanding of B cells and the generation of antibodies in natural infection and/or through vaccination remains limited [18]. Given that research on B cells during pregnancy remains in its early stages, more investigations into antibody function, B cells, and vaccine-induced B cell responses during pregnancy are critically needed [18, 59].

There are several limitations to this study. First, the neutralization assay was performed using pseudoviruses rather than authentic SARS-CoV-2 variants. Additionally, the neutralizing assay was conducted mainly against SARS-CoV-2 D614G, Alpha, Beta, Gamma, Delta, BA.1, BA.2.12.1, and BA.4/5. Another limitation was the relatively small sample size and the fact that the pregnant women were immunized in the first trimester of pregnancy. Given the immunological changes that occur throughout pregnancy [1, 67], further studies involving a larger number of pregnant women vaccinated across all trimesters are necessary.

In summary, this study is the first in which the potency of memory B cell and antibody responses was analyzed at the molecular level in pregnant women. While pregnant women demonstrated a robust ability to generate serological responses to the inactivated COVID-19 vaccine, notable differences in the potency of vaccine-induced memory B cells and vaccine-specific mAbs between pregnant and non-pregnant women were observed. These findings will be informative for vaccine design and updating and the selection and optimization of vaccination strategies for pregnant women.

## Supplementary Information

Supplementary Material 1.

## Data Availability

The original contributions presented in the study are included in the article/Supplementary Material. Further inquiries can be directed to the corresponding authors.
