# Supplementary material for: Immunogenicity and memory B-cell potency induced by an inactivated COVID-19 vaccine in pregnant women
Source: Mol Med. 2025 Sep 26;31:297. doi: 10.1186/s10020-025-01357-5 (PMC12465604; doi:10.1186/s10020-025-01357-5)
Supplement: Supplementary file 1 — Supplementary Material 1. [file 10020_2025_1357_MOESM1_ESM.docx]

**Supplementary Information for**

**Immunogenicity and Memory B-cell Potency Induced by an Inactivated COVID-19 Vaccine in Pregnant Women**

**Wen et al.**

**This file includes:**

**Supplementary Tables S1 to S2**

**Supplementary Figs. S1 to S14**

**Table S1 Characteristics and plasma neutralizing activity of pregnant women and non-pregnant women**

| Characteristics | Pregnant women  (n=4) | Non-pregnant women  (n=11) | Total  (n=15) |
| --- | --- | --- | --- |
| Age, years | 30.25±4.57 | 31.64±4.23 | 31.27±4.20 |
| D614G, n (%) | 4 (100.0) | 10 (90.9) | 14 (93.3) |
| Alpha, n (%) | 4 (100.0) | 9 (81.8) | 13 (86.7) |
| Beta, n (%) | 1 (25.0) | 5 (45.5) | 6 (40.0) |
| Gamma, n (%) | 4 (100.0) | 9 (81.8) | 13 (86.7) |
| Delta, n (%) | 3 (75.0) | 5 (45.5) | 8 (53.3) |
| BA.1, n (%) | 0 (0.0) | 0 (0.0) | 0 (0.0) |
| BA.2.12.1, n (%) | 0 (0.0) | 1 (9.1) | 1 (6.7) |
| BA.4/5, n (%) | 0 (0.0) | 1 (9.1) | 1 (6.7) |

Continuous data are presented as the means ± standard deviations, and categorical data are presented as numbers (percentages).

**Table S2 Isolation of severe acute respiratory syndrome coronavirus 2 (SARS-CoV-2) spike (S)-specific monoclonal antibodies (mAbs) from five participants**

| Characteristics | Donor 1 | Donor 2 | Donor 3 | Donor 4 | Donor 5 |
| --- | --- | --- | --- | --- | --- |
| Age, years | 28 | 25 | 34 | 31 | 30 |
| Group | Pregnant | Pregnant | Non-pregnant | Non-pregnant | Non-pregnant |
| Starting PBMCs | ~9.0×10^6^ | ~9.0×10^6^ | ~9.0×10^6^ | ~9.0×10^6^ | ~9.0×10^6^ |
| SARS-CoV-2 S-specific B cells, n | 16 | 25 | 16 | 34 | 34 |
| Cloned antibodies, n | 11 | 14 | 11 | 17 | 14 |
| SARS-CoV-2 S-specific mAbs, n | 11 | 11 | 11 | 17 | 11 |

Sixteen to 34 SARS-CoV-2 S-specific B cells from each participant were sorted into individual wells, with their Ig gene transcripts recovered by single-cell polymerase chain reaction (PCR). Subsequently, 11 to 17 cognate heavy and light chain pairs from each donor were cloned into the IgG1 vector and expressed as mAbs in ExpiCHO cells. In summary, a total of 61 SARS-CoV-2 S-specific mAbs, 11 to 17 from each participant, were obtained. PBMCs, peripheral blood mononuclear cells.


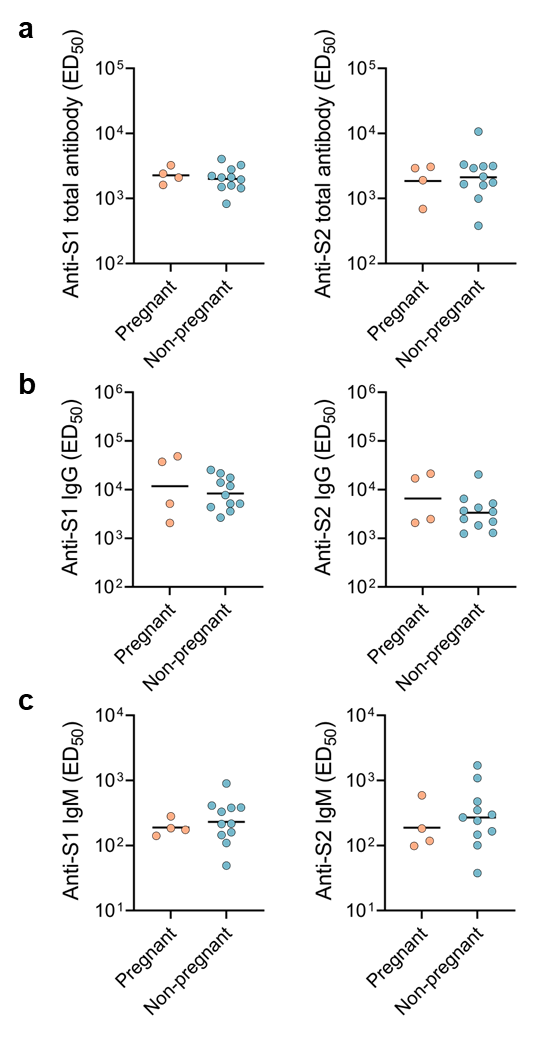


**Fig. S1. The levels of total antibody, IgG antibody, and IgM antibody against the S1 and S2 regions of the SARS-CoV-2 spike protein in the participants. (a)** Levels of total antibody (half-maximal effective dilution, ED_50_) against the S1 and S2 proteins in plasma. **(b)** Levels of IgG antibody (ED_50_) against the S1 and S2 proteins in plasma. **(c)** Levels of IgM antibody (ED_50_) against the S1 and S2 proteins in plasma.


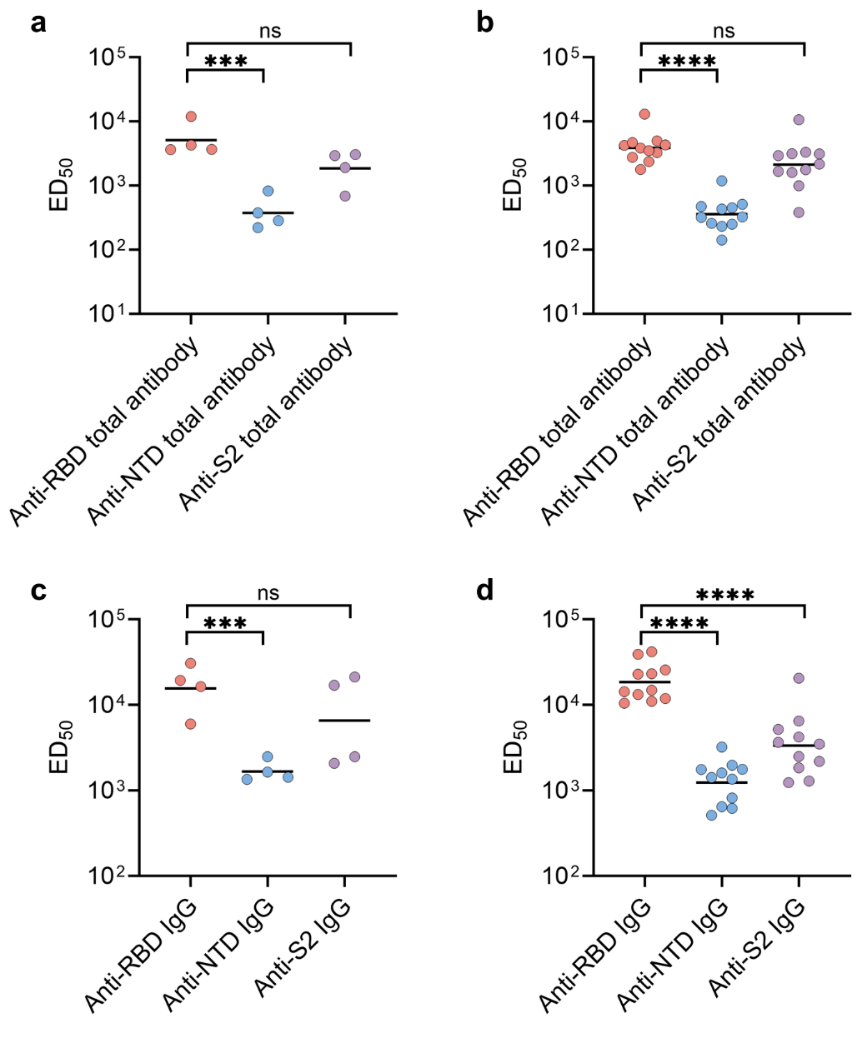


**Fig. S2. Total antibody and IgG antibody levels against the receptor-binding domain (RBD), N-terminal domain (NTD), and S2 of the SARS-CoV-2 spike (S) protein in the participants.** **(a)** The levels of total antibody against the RBD (anti-RBD total antibody), NTD (anti-NTD total antibody), and S2 (anti-S2 total antibody) in pregnant women (n = 4). **(b)** The levels of anti-RBD total antibody, anti-NTD total antibody, and anti-S2 total antibody in non-pregnant women (n = 11). **(c)** The levels of IgG antibody against RBD (anti-RBD IgG), NTD (anti-NTD IgG), and S2 (anti-S2 IgG) in pregnant women (n = 4). **(d)** The levels of anti-RBD IgG, anti-NTD IgG, anti-S2 IgG in non-pregnant women (n = 11). The difference was calculated via an unpaired t test after logarithmic transformation. ns: no significant difference; *: p<0.05; **: p<0.01; ***: p<0.001; ****: p<0.0001.


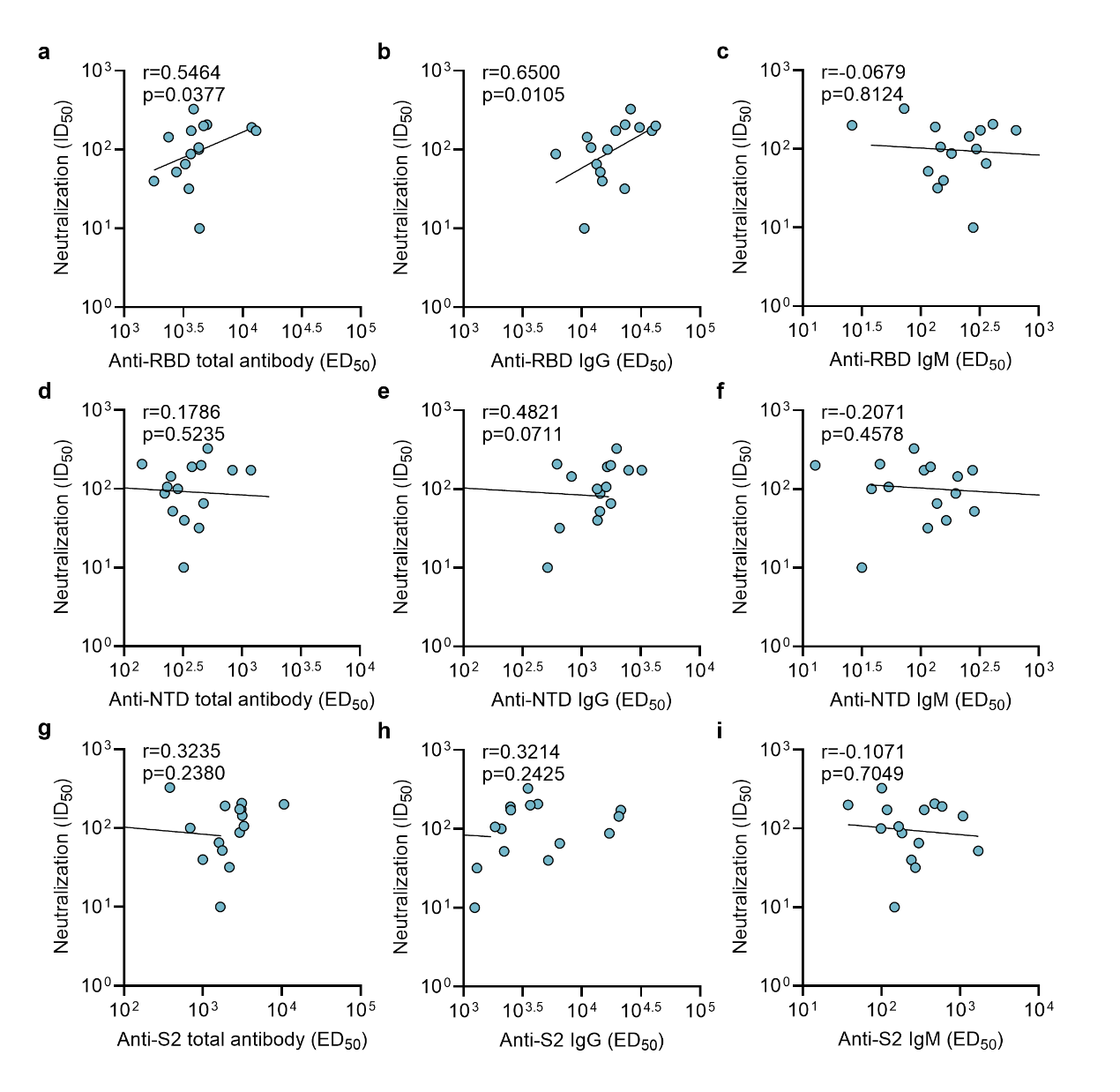


**Fig. S3. Relationships between enzyme-linked immunosorbent assay (ELISA)-binding titers and neutralizing titers in the plasma (n = 15).** (a) The relationship between anti-receptor-binding domain (RBD) total antibody and neutralizing titers. (b) The relationship between anti-RBD IgG and neutralizing titers. (c) The relationship between anti-RBD IgM and neutralizing titers. (d) The relationship between anti-N-terminal domain (NTD) total antibody and neutralizing titers. (e) The relationship between anti-NTD IgG and neutralizing titers. (f) The relationship between anti-NTD IgM and neutralizing titers. (g) The relationship between anti-S2 total antibody and neutralizing titers. (h) The relationship between anti-S2 IgG and neutralizing titers. (i) The relationship between anti-S2 IgM and neutralizing titers. Correlations were determined by the Spearman rank correlation test. *r* and p values are indicated.


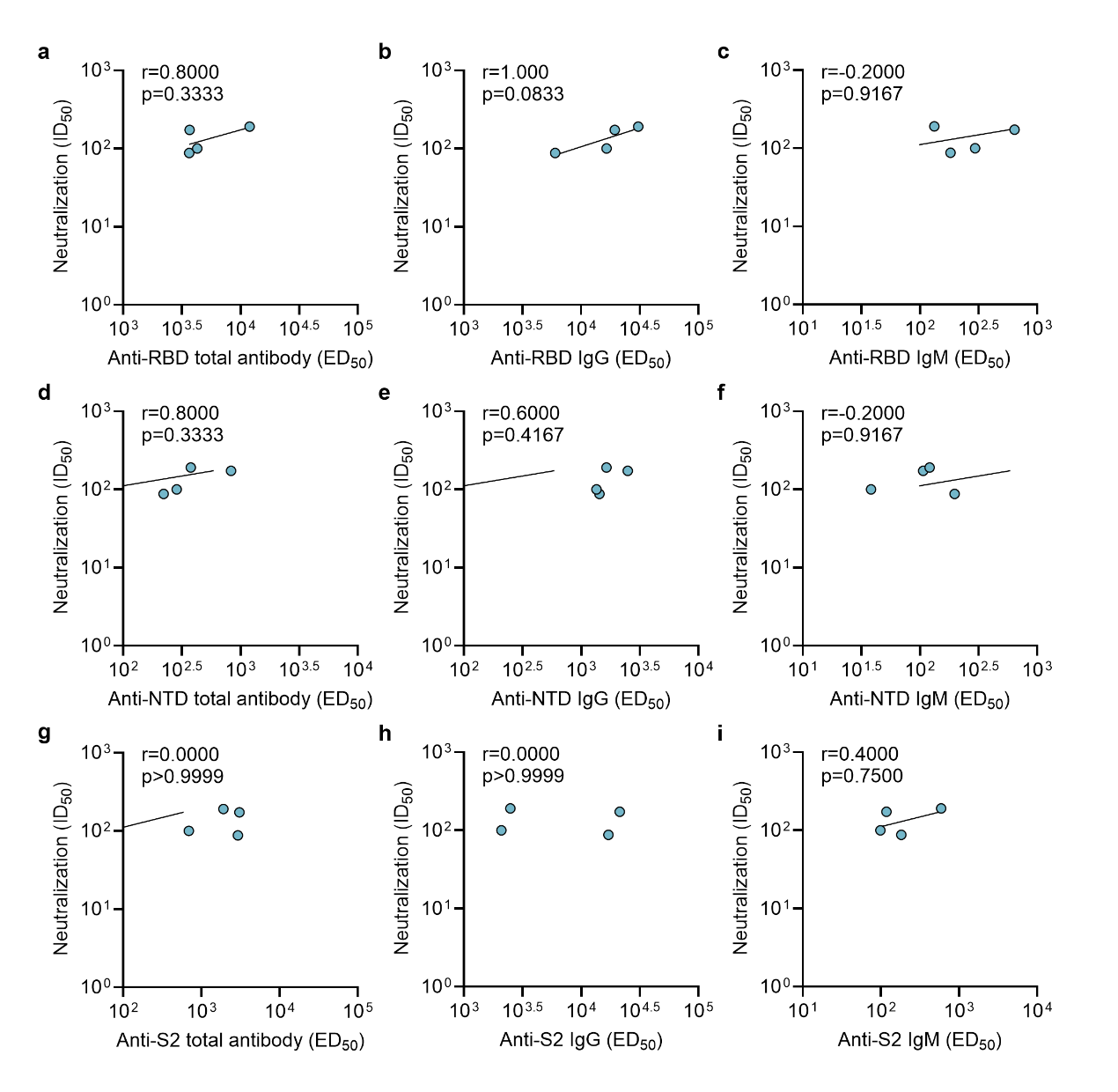


**Fig. S4. Relationships between enzyme-linked immunosorbent assay (ELISA)-binding titers and neutralizing titers in the plasma from pregnant women (n = 4).** (a) The relationship between anti-receptor-binding domain (RBD) total antibody and neutralizing titers. (b) The relationship between anti-RBD IgG and neutralizing titers. (c) The relationship between anti-RBD IgM and neutralizing titers. (d) The relationship between anti-N-terminal domain (NTD) total antibody and neutralizing titers. (e) The relationship between anti-NTD IgG and neutralizing titers. (f) The relationship between anti-NTD IgM and neutralizing titers. (g) The relationship between anti-S2 total antibody and neutralizing titers. (h) The relationship between anti-S2 IgG and neutralizing titers. (i) The relationship between anti-S2 IgM and neutralizing titers. Correlations were determined by the Spearman rank correlation test. *r* and p values are indicated.


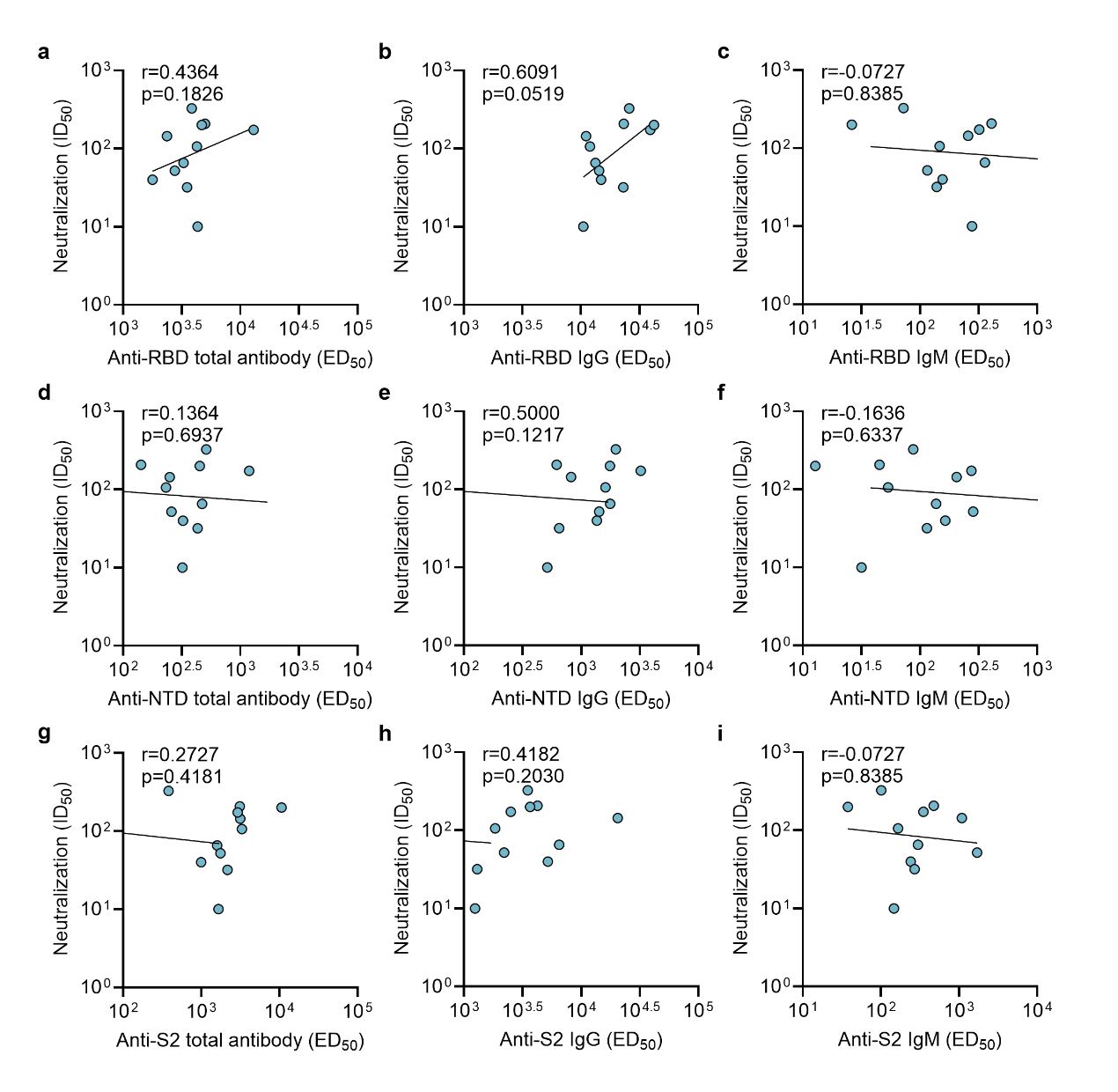


**Fig. S5. Relationships between enzyme-linked immunosorbent assay (ELISA)-binding titers and neutralizing titers in the plasma from non-pregnant women (n = 11).** (a) The relationship between anti-receptor-binding domain (RBD) total antibody and neutralizing titers. (b) The relationship between anti-RBD IgG and neutralizing titers. (c) The relationship between anti-RBD IgM and neutralizing titers. (d) The relationship between anti-N-terminal domain (NTD) total antibody and neutralizing titers. (e) The relationship between anti-NTD IgG and neutralizing titers. (f) The relationship between anti-NTD IgM and neutralizing titers. (g) The relationship between anti-S2 total antibody and neutralizing titers. (h) The relationship between anti-S2 IgG and neutralizing titers. (i) The relationship between anti-S2 IgM and neutralizing titers. Correlations were determined by the Spearman rank correlation test. *r* and p values are indicated.

**
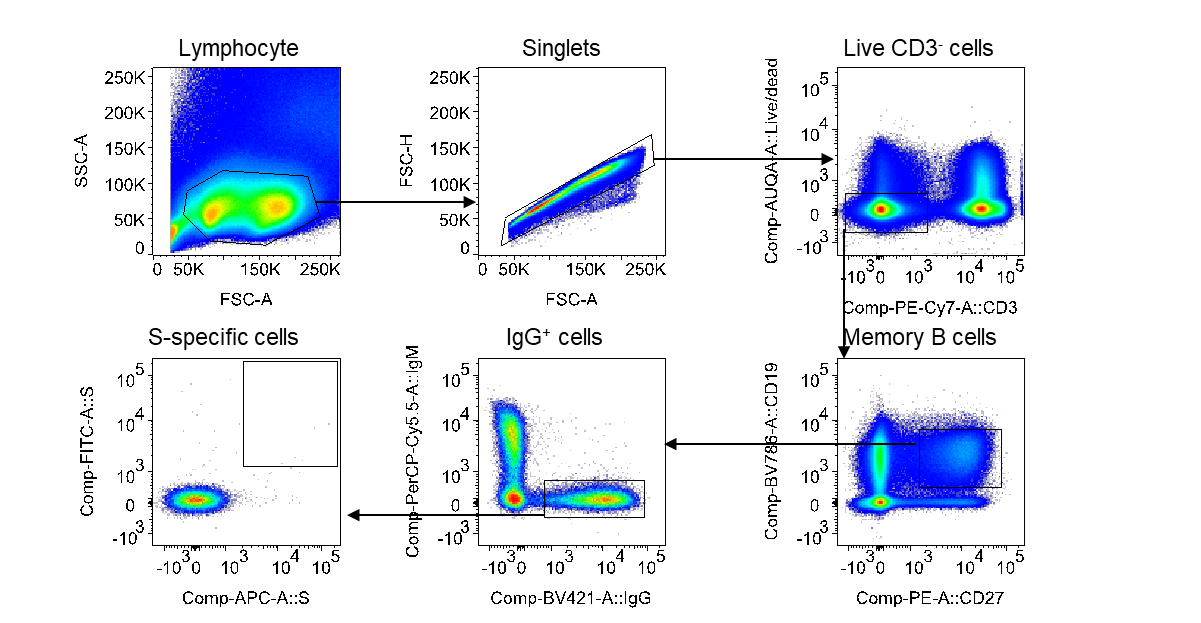
**

**Fig. S6. Representative gating strategy for IgG+ SARS-CoV-2 spike (S)-specific memory B cells.** Live CD3-CD19+CD27+IgM-IgG+S+ cells were defined as IgG+ SARS-CoV-2 S-specific memory B cells and were single-cell sorted for antibody cloning. SSC-A, side scatter area; FSC-H, forward scatter height; FSC-A, forward scatter area.


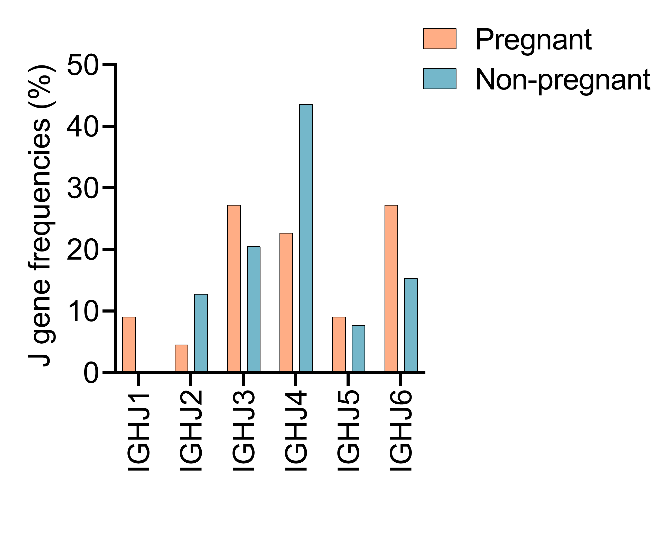


**Fig. S7. Distribution of joining (J) germline genes of the heavy chain.**


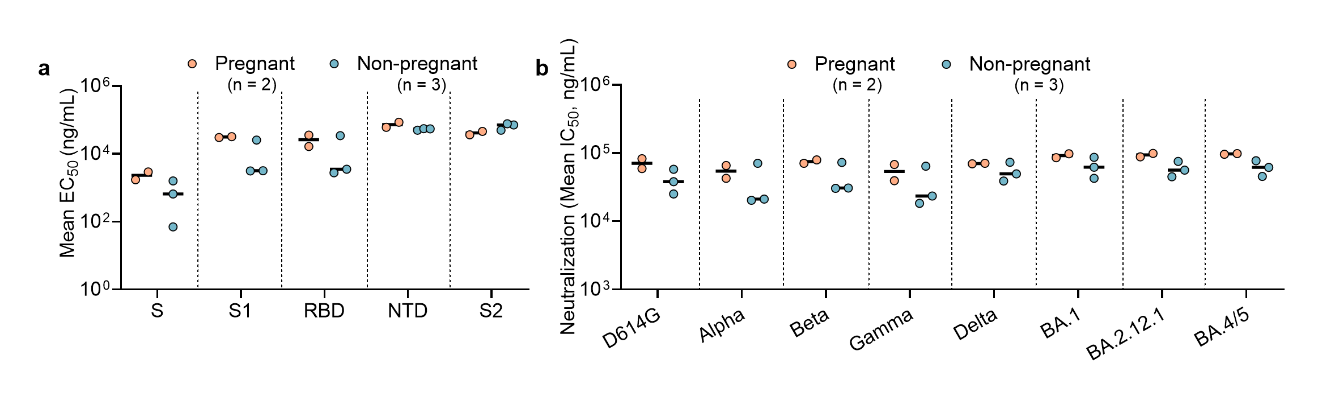


**Fig. S8. Mean functional activities of severe acute respiratory syndrome coronavirus 2 (SARS-CoV-2) spike(S)-specific monoclonal antibodies (mAbs) in each participant.** **(a)** Mean antibody-binding activity (half-maximal effective concentration, EC_50_) of the SARS-CoV-2 S, S1, receptor-binding domain (RBD), N-terminal domain (NTD), and S2 protein in each participant. Each dot represents a participant. **(b)** Mean antibody-neutralizing activity (half-maximal inhibitory concentration, IC_50_) against SARS-CoV-2 D614G, Alpha, Beta, Gamma, Delta, BA.1, BA.2.12.1, and BA.4/5 in each participant. Each dot represents a participant.


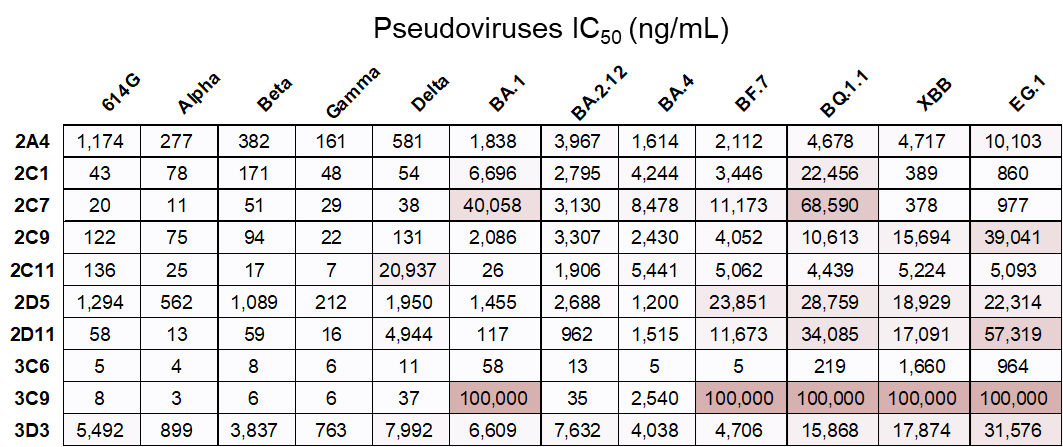


**Fig. S9. Neutralization profile of severe acute respiratory syndrome coronavirus 2 (SARS-CoV-2) pseudoviruses for 10 purified monoclonal antibodies from non-pregnant women.** The number in the box indicates the half-maximal inhibitory concentration (IC_50_) value. The color gradient indicates IC_50_ values ranging from 0 (white) to 100,000 ng/mL. Antibodies with IC_50_ values above 100,000 ng/mL were plotted as 100,000 ng/mL.


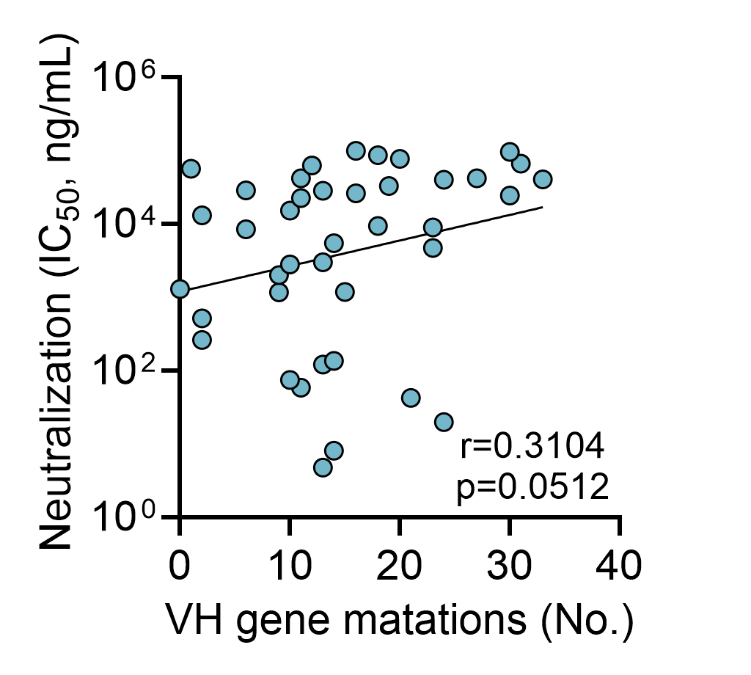


**Fig. S10. Relationships between somatic hypermutation (SHM) and severe acute respiratory syndrome coronavirus 2 (SARS-CoV-2) neutralizing activity.** The neutralization half-maximal inhibitory concentration (IC_50_) is plotted against the number of heavy-chain variable (VH) nucleotide substitutions for each monoclonal antibody (mAb). Notably, since non-neutralizing mAbs were excluded from the statistical analysis, they are not shown in this plot. Forty mAbs are shown in this plot. Each dot represents a single mAb. Spearman rank correlation analysis was used to determine the correlation between SHM and neutralization activity. The p and r values are shown.


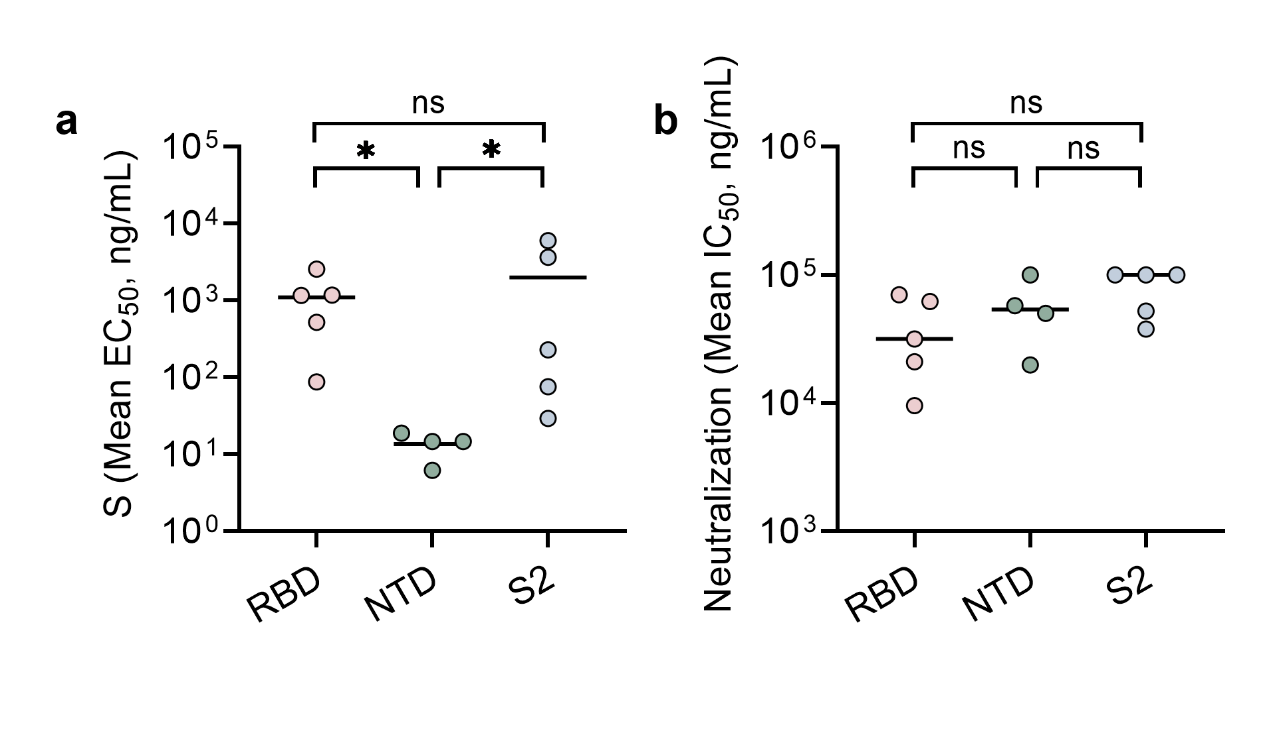


**Fig. S11. Mean binding activity (a) and neutralizing activity (b) per participant of monoclonal antibodies (mAbs) targeting RBD, NTD, and S2.** Each dot represents a per participant. The NTD-recognizing mAbs were observed in four per participant, thus four dots are shown for the NTD-recognizing mAbs. Differences were calculated via the Mann‒Whitney U test. ns, no significant difference; *p < 0.05.


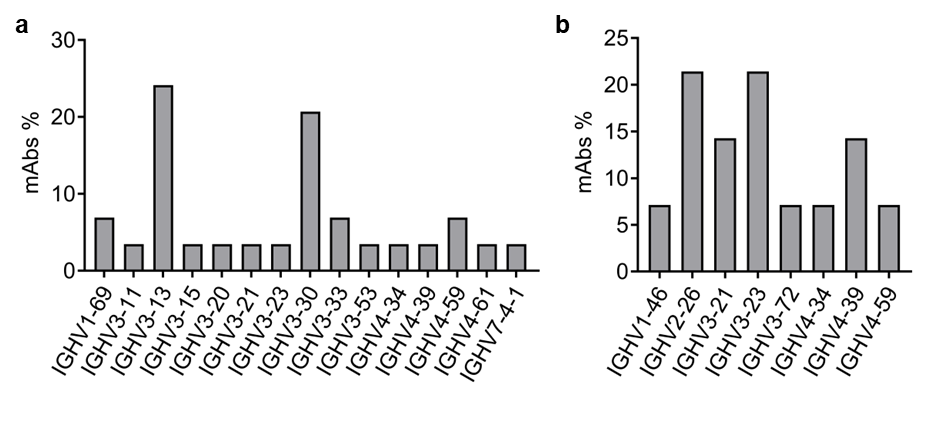


**Fig. S12. Distribution of the variable genes of the heavy-chain (VH) germline genes of monoclonal antibodies (mAbs) recognizing the receptor-binding domain (RBD) (a) and N-terminal domain (NTD) (b).**


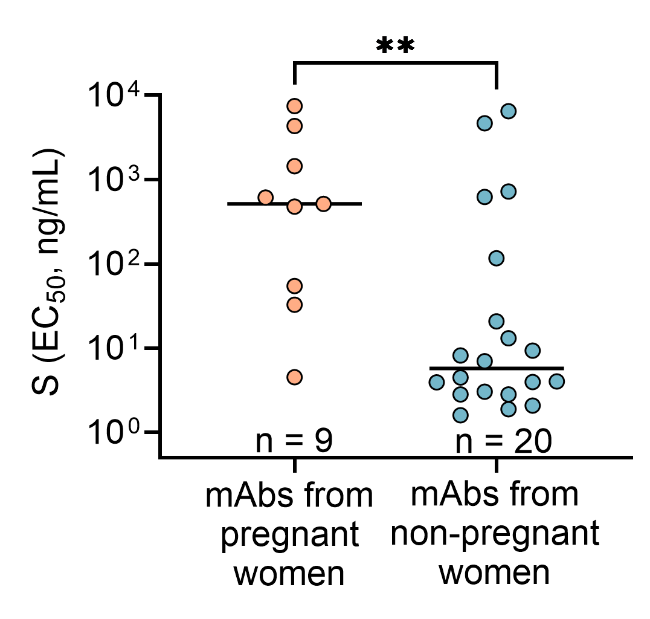


**Fig. S13. Binding activity of receptor-binding domain-recognizing monoclonal antibodies (mAbs) from pregnant women and mAbs from non-pregnant women.** Each dot represents a single mAb. Difference was calculated via the Mann‒Whitney U test. **: p<0.01.


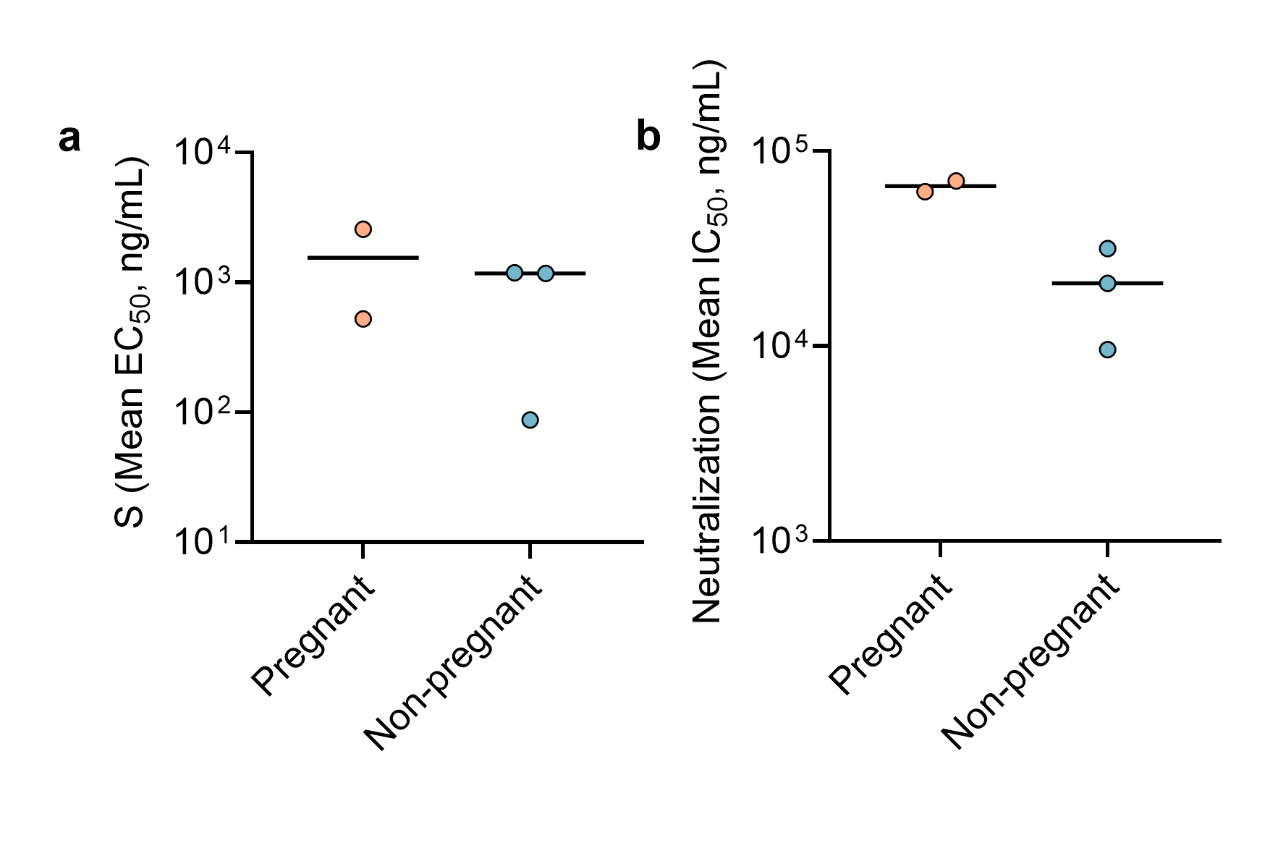


**Fig. S14. Mean binding activity (a) and neutralizing activity (b) of receptor-binding domain-recognizing monoclonal antibodies in each participant.** Each dot represents a participant.
